# Dr. Jennie Mary Turner

**Published:** 1914-11

**Authors:** 


Dr. Jennie Mary Turner, Mich., 1879, died at the Rochester General Hospital, September 17, aged 63. She was a practitioner of Newark, N. Y. for 30 years.

